# Study on Oil Displacement Mechanism of Betaine/Polymer Binary Flooding in High-Temperature and High-Salinity Reservoirs

**DOI:** 10.3390/molecules30051145

**Published:** 2025-03-03

**Authors:** Xiuyu Zhu, Qun Zhang, Changkun Cheng, Lu Han, Hai Lin, Fan Zhang, Jian Fan, Lei Zhang, Zhaohui Zhou, Lu Zhang

**Affiliations:** 1 Oil & Gas Technology Research Institute, PetroChina Qinghai Oilfield Company, Dunhuang 736202, China; zhuxlqh@petrochina.com.cn (X.Z.); cckqh@petrochina.com.cn (C.C.); linhaiqh@petrochina.com.cn (H.L.); 2 State Key Laboratory of Enhanced Oil & Gas Recovery, PetroChina Research Institute of Petroleum Exploration & Development, Beijing 100083, China; zhangqun1980@petrochina.com.cn (Q.Z.); hanlu1991@petrochina.com.cn (L.H.); zhangfan902@petrochina.com.cn (F.Z.); fanjian@petrochina.com.cn (J.F.); 3 Key Laboratory of Photochemical Conversion and Optoelectronic Materials, Technical Institute of Physics and Chemistry, Chinese Academy of Sciences, Beijing 100190, China; zhanglei@mail.ipc.ac.cn

**Keywords:** betaine, polymer, bulk viscosity, binary flooding, oil displacement mechanism

## Abstract

As an efficient and economical method to enhance oil recovery (EOR), it is very important to explore the applicability of chemical flooding under harsh reservoir conditions, such as high temperature and high salinity. We designed microscopic visualization oil displacement experiments to comprehensively evaluate the oil displacement performance of the zwitterionic surfactant betaine (BSB), a temperature- and salinity-resistant hydrophobically modified polymer (BHR), and surfactant–polymer (SP) binary systems. Based on macroscopic properties and microscopic oil displacement effects, we confirmed that the BSB/BHR binary solution has the potential to synergistically improve oil displacement efficiency and quantified the reduction in residual oil and oil displacement efficiency within the swept range. The experimental results show that after water flooding, a large amount of residual oil remains in the porous media in the form of clusters, porous structures, and columnar formations. After water flooding, only slight emulsification occurred after the injection of BSB solution, and the residual oil could not be activated. The injection of polymer after water flooding can expand the swept range to a certain extent. However, the distribution of residual oil in the swept range is similar to that of water flooding, and the oil washing efficiency is low. The SP binary flooding process can expand sweep coverage and effectively decompose large oil clusters simultaneously. This enhances the oil washing efficiency within the swept area and can significantly improve oil recovery. Finally, we obtained the microscopic oil displacement mechanism of BSB/BHR binary system to synergistically increase the swept volume and effectively activate the residual oil after water flooding. It is the result of the combined action of low interfacial tension (IFT) and suitable bulk viscosity. These findings provide critical insights for optimizing chemical flooding strategies in high-temperature and high-salinity reservoirs, significantly advancing EOR applications in harsh environments.

## 1. Introduction

As a national strategic energy source, oil provides an important guarantee for economic development and daily life. With the increasing exploration and development efforts in oil and gas fields, unconventional resources will become an important strategic replacement for oil and gas development [1,2,3]. The reserves of high-temperature and high-salinity reservoirs are abundant, but the overall oil recovery is low. Therefore, improving the enhanced oil recovery (EOR) of such reservoirs has become a research hotspot [4]. The high reservoir temperature (>85 °C) and salinity (>100,000 ppm) of reservoirs, which are usually significantly higher compared to conventional reservoirs, are emerging as a key driver of EOR because they can determine the type of oil displacement agent selection and, at the same time, can affect the effectiveness of the displacement process [5,6].

As a common way to improve oil recovery efficiency, chemical flooding has a certain potential for EOR in harsh reservoirs [7]. One method is to inject surfactant solution. In particular, the surfactant solution reduces the oil–water interfacial tension (IFT), and reduces the capillary resistance, thus contributing to reducing the water injection pressure and enhancing the effectiveness of oil recovery [8,9]. For example, Lv and Zhang et al. formed oil-in-water (O/W) emulsion through in situ emulsification and viscosity enhancement of betaine solution, which effectively improved the development effect of the reservoir. The plugging effect is used to achieve the profile control effect in favor of the expansion of sweep range and improves the recovery rate within the sweep range under low pressure conditions [10,11]. Nevertheless, with the increase in heterogeneity, coupled with the large difference in the water–oil mobility ratio, the seepage velocity of surfactant solution alone is too fast. As a result, the dominant channel is generated, and the in situ emulsification ability is weakened, which is not much different from the effect of water flooding [12]. Another method is polymer flooding. A typical application is that the injection of water-soluble polymers (partially hydrolyzed and hydrophobically modified polyacrylamide) to increase the bulk viscosity and slow down the migration rate of the displacement fluid, thereby effectively expanding the sweep range [13,14]. However, under the combined actions of high temperature and high salinity, the polymer may be affected by salinity, temperature, pH value, injection time, and so on. The ability to increase viscosity is weakened or flocculated, which makes the application failing to achieve the expected goal [15,16].

It is considered that the ideal method is surfactant–polymer (SP) binary oil displacement system, where the critical oil displacement mechanisms mainly comprise two aspects. On the one hand, it slows down the seepage velocity of the surfactant solution, avoids the occurrence of water channeling, increases the in situ emulsification effect, and improves the oil washing efficiency. On the other hand, the introduction of polymers increases fluid viscosity, increases flow resistance, and effectively improves the expansion effect [17,18]. The synergy of the two can reduce IFT, enhance emulsion stability, and improve rheological properties, thereby improving displacement effects compared with the surfactant flooding or polymer flooding alone [19]. Numerous scholars have also confirmed the effectiveness of the binary flooding [20,21]. Nevertheless, to date, the improvement effect of the conventional SP profile control system has been limited. In addition, the surfactants and polymers used need to be changed due to high temperature and salinity limits. Therefore, SP systems suitable for high-temperature and high-salinity reservoirs must be screened for temperature- and salinity-resistant surfactants and polymers. And both must work together for excellent oil displacement effect without compromising their respective interfacial properties. This also means that it is necessary to not only screen the excellent SP binary formulas but also to explore the corresponding oil displacement mechanism so as to provide guidance for the deep development of high-temperature and high-salinity reservoirs.

Restricted by harsh reservoir conditions, a temperature- and salinity-resistant betaine and a hydrophobically modified polymer were introduced to construct a binary oil displacement system in this study. The polymer is synthesized by copolymerization of polyacrylamide as the main chain and introduction of strong polarity and salinity-resistant functional monomers. Inspired by the detailed reports of the IFT reduction, emulsification characteristics, droplet coalescence, rheological properties, and oil displacement performance of betaine solutions [22,23], in this paper, the microscopic visualization oil displacement method is used to evaluate the oil displacement effect of betaine solution, hydrophobic modified polyacrylamide solution, and their combinations in porous media. Furthermore, the influences of macroscopic properties such as IFT, bulk viscosity, and rheological properties on displacement effect and seepage characteristics are also combined. The aim is to clarify the microscopic mechanism of binary flooding at pore scale, and to provide method reference and technical guidance for chemical flooding of high-temperature and high-salinity reservoirs.

## 2. Results and Discussion

### 2.1. Oil-Water Interfacial Tensions

**Figure 1:**
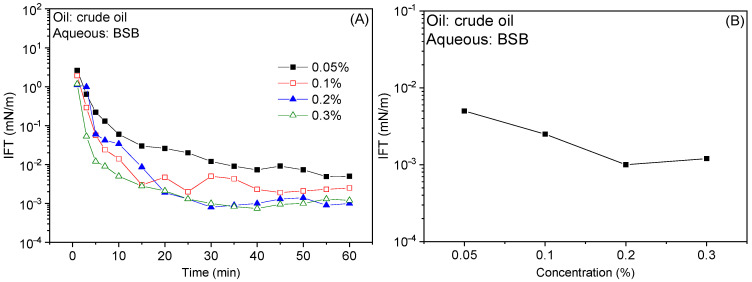
Effect of concentration on dynamic (**A**) and equilibrium (**B**) IFTs between BSB solution and crude oil.

Figure 1 shows the dynamic and equilibrium IFTs curves of betaine zwitterionic surfactant BSB and crude oil as a function of surfactant concentration. In Figure 1A, the dynamic IFT curve between BSB solution and crude oil shows an “L” shape, which gradually decreases to the platform value with the increase in time and reaches an ultra-low level (10^−3^ mN/m). Meanwhile, the IFT gradually decreases with increasing BSB concentration. In particular, there is a synergistic effect between the betaine solution and the active components in the crude oil, and a tight mixed adsorption film is formed at the oil/water interface, which reduces the IFT to ultra-low level [24,25]. In general, BSB solution has good interfacial activity within the experimental concentration range (0.05–0.3%). When the solution concentration is greater than 0.2%, the equilibrium value of the IFT can reach the platform in Figure 1B. Therefore, in the subsequent experiments, the concentration of the surfactant was 0.3% as the research basis to ensure good interfacial activity.

**Figure 2:**
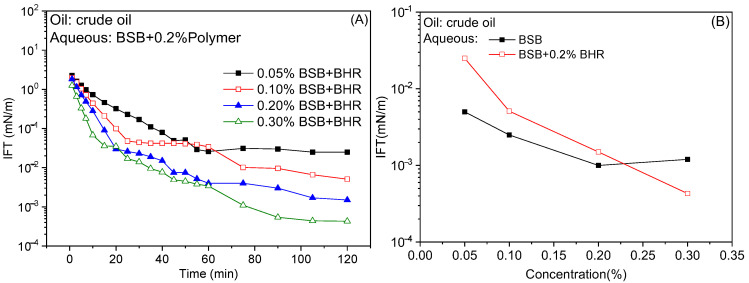
Effect of betaine concentration on dynamic (**A**) and equilibrium (**B**) IFTs between binary solutions and crude oil.

For the SP system, fixed the polymer concentration (0.2%), the IFT of the system gradually decreased with the increase in surfactant concentration, and reached the equilibrium value within 120 min, as shown in Figure 2A. Since the interfacial film strength of betaine is high and the addition of polymers with low interfacial activity and large molecular size cannot destroy the interfacial film, the IFT of the SP system is still low [23]. Comparing the IFT of the BSB and SP systems with the concentration (Figure 2B), when the BSB concentration is between 0 and 0.2%, the IFT of the surfactant solution alone is lower. When the concentration is greater than 0.2%, the BSB has reached the platform value and no longer changes, but the SP system can produce a synergistic effect to further reduce it. This is because at low surfactant concentrations, BSB and the hydrophobic segments of the polymer form aggregates through hydrophobic interactions, which reduces the number of monomeric surfactant molecules at the interface and increases the IFT of the SP system [26]. Correspondingly, at high surfactant concentrations, as the surfactant molecules interacting with the hydrophobic block of the polymer molecule reach saturation, the excess molecules can still form a tightly arranged interfacial film. At this time, the synergistic effect between polymer and surfactant makes the IFT of SP system even lower [27].

**Figure 3:**
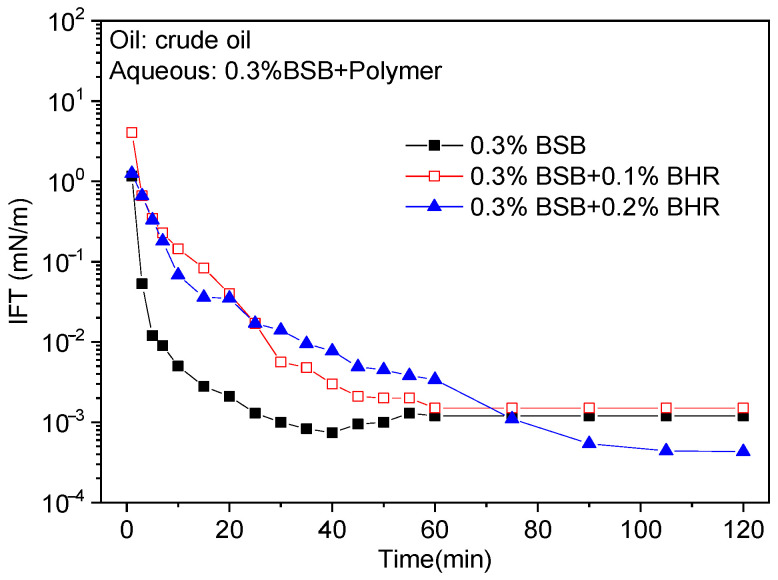
Effect of polymer concentration on dynamic IFTs between binary solutions and crude oil.

The effect of polymer concentration on the IFTs between surfactant solutions and crude oil were investigated by fixing a relatively higher surfactant concentration (0.3%), as shown in Figure 3. Elevated viscosity increases with polymer concentration, causing mass transfer difficulties at the interface and slowing down the adsorption rate of surfactant to the oil/water interface. Specifically, the dynamic IFT decreases more slowly after the addition of the polymer, but the equilibrium IFT can still be stabilized at the order of 10^−3^ mN/m. In addition, as mentioned above, the synergistic effect is evident as the polymer concentration increases with a subsequent further decrease in IFT.

### 2.2. Bulk Viscosity

In the process of chemical flooding, polymer flooding is a favorable choice in many reservoirs by controlling the fluidity of the displacement fluid inside the reservoir to ensure higher recovery [28]. Among them, the bulk viscosity parameter is considerably crucial to the success of viscoelastic polymer flooding applications [29,30]. Maintaining the viscosity of the polymer under high-temperature and high-salinity reservoir conditions can provide a favorable guarantee for the flooding performance of the subsequent SP binary flooding.

**Figure 4:**
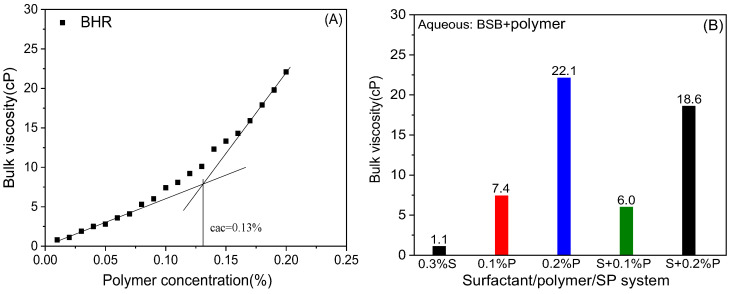
(**A**) Concentration dependence of the bulk viscosity of polymer solution; (**B**) bulk viscosity of surfactant/polymer/SP binary system.

Figure 4A illustrates the viscosity increase ability of BHR under high-salinity conditions. As the polymer concentration increases, the viscosity increases exponentially. When the concentration was 0.2%, the viscosity increased to 22.1 cP, reflecting certain viscosity enhancement and salinity resistance [31]. This is because, as the concentration increases, the hydrophobically modified polymer molecules can form a three-dimensional network structure through interactions between the hydrophobic blocks, resulting in a nonlinear increase in viscosity. The concentration of the polymer that begins to form a network structure is called the critical association concentration (cac), which can be obtained from the viscosity concentration curve. The cac of BHR studied in this paper is about 0.13%.

Figure 4B shows the viscosity data of S/P/SP solutions. For the SP system, the viscosity is slightly lower than that of the individual polymer. The reason for the decrease in viscosity is the destruction of the polymer network structure. Generally, with the increase in surfactant concentration, the hydrophobic interaction between the polymer hydrophobic block and the surfactant molecules in the solution gradually increases. As a result, the network structure associated with different polymer molecules gradually disintegrated, forming aggregates of macromolecular polymers and small molecular surfactants, which greatly reduced the bulk viscosity. Interestingly, the viscosity of the betaine complexed with the hydrophobically modified polymer system is only slightly reduced, and still able to meet the viscosity increase requirements of oil displacement. The multifunctional groups in betaine molecules can interact with multiple polymer molecules, playing an associative role and causing less disruption to the network structure [23].

### 2.3. Oil Displacement Effect

In this work, we conducted five sets of microscopic visualization oil displacement experiments to evaluate the effects of different chemical slugs (S/P/SP system) on the state and oil displacement efficiency of residual oil after water flooding. We investigated the effects of several parameters on oil displacement efficiency, including oil/water IFT, polymer concentration, and polymer slug viscosity. In the experiment, the crude oil in the same chip model was flooded with 1 PV water flooding, and then the same amount (2 PV) of chemical slug flooding was performed. All displacement fluids were injected at the same flow rate. Afterwards, the segmented images after displacement were analyzed to obtain the oil displacement efficiency, sweep efficiency, oil displacement efficiency within the sweep range, and oil displacement efficiency in the expanded sweep area for each group of experiments.

Table 1 presents the oil displacement efficiency data of S/P/SP solutions. For water flooding, only a small amount of crude oil is displaced due to the high viscosity of crude oil, high seepage resistance, and high water/oil mobility ratio, resulting in low oil displacement efficiency (about 40%) [32]. After water flooding, continued injection of surfactant solution (0.3% S) showed minimal improvement in oil displacement efficiency. Because the viscosity of the surfactant solution is almost equivalent to that of water, the solution flows along the dominant channel formed by water flooding, which cannot expand the sweep range, and the oil displacement efficiency is only less than 1.0% higher than that of water flooding. For polymer flooding, the bulk viscosity and oil displacement efficiency rise with the increasing polymer concentration. Although the increase is limited (5.3–11.9%), it also shows that the effect of viscosity increase occupies a major position in the mechanism of polymer EOR. After the injection of 0.3% S + 0.1% P solution, the oil displacement efficiency of the system has been significantly improved (13.3%). This result also indicates that although the system has ultra-low IFT, the increase in oil displacement efficiency is limited due to insufficient viscosity. Ultimately, after the injection of 0.3% S + 0.2% P solution, the system has both ultra-low IFT and enough viscosity, and the oil displacement efficiency is greatly enhanced (31.3%). Therefore, based on the oil displacement efficiency data, it is preliminarily speculated that BSB and BHR have a synergistic effect. Meanwhile, the central oil displacement mechanism of the SP system is mainly based on low IFT and viscosity increase, which will be researched in the following experiments.

**Figure 5:**
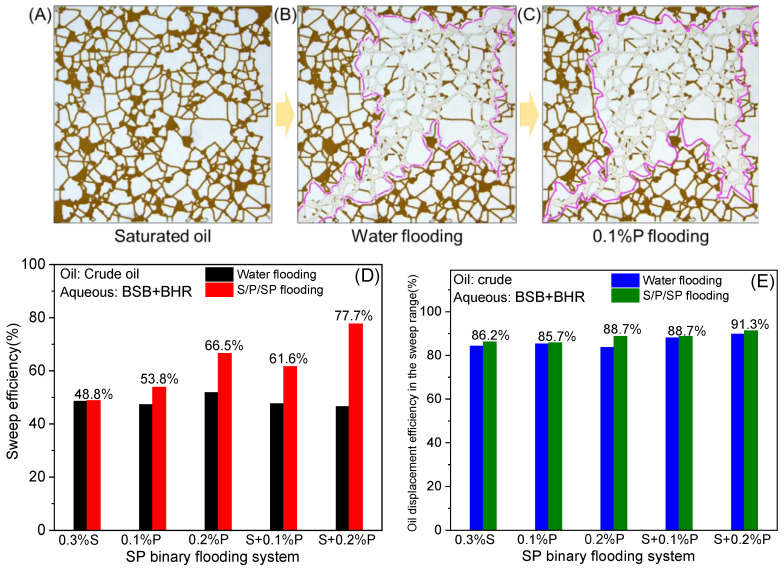
(**A**) Saturated oil state; (**B**,**C**) displacement effect of formation water and 0.1% P solution, respectively; (**D**,**E**) the sweep efficiency and the oil displacement efficiency within the sweep range calculated by the final oil displacement results.

Taking the 0.1% polymer solution as an example, Figure 5A–C show the saturated oil state, the residual oil after water flooding, and polymer flooding, respectively. The sweep range (pink outline) is defined by taking the edge of completely un-started residual oil as the boundary line in Figure 5B,C so as to further discuss the synergistic mechanism of SP flooding. Figure 5D,E present the overall sweep efficiency and the oil displacement efficiency within the sweep range. The sweeping effect of BSB solution alone is relatively poor, only 48.8%, as shown in Figure 5D. The reason for the poor sweep effect is that the oil/water interface film of BSB has high strength, the emulsion deformation ability is poor, and the solution has a weak ability to mobilize the residual oil after water flooding [33]. The oil displacement efficiency data in Table 1 indicate that the displacement effect of BSB is not good, but its oil displacement efficiency within the sweep range is not much different from that of other systems. From the sweep efficiency (48.8%) data, it can be seen that the sweep volume is not expanded based on water flooding, and only a small amount of residual oil within the water flooding range is mobilized (Figure 5E).

After the polymer solution is injected alone, it gradually fills the pores after water flooding and then is sheared by the porous media structure during the flow process. The viscoelastic effect increases the macroscopic flow resistance, improves the mobility ratio, and expands the sweep range. However, it has little effect on the oil washing capacity within the sweep range. The fluid viscosity, seepage resistance, and sweep range increased after the combination of surfactant and polymer (0.3% S + 0.1% P), but here was also no significant increase in the oil displacement efficiency within the sweep range. With the further increase in polymer concentration, 0.3% S + 0.2% P injection, the oil displacement efficiency within the sweep range is slightly improved. Overall, the uplift is limited. By comparing Figure 5B,C, it can be found that all fluids can no longer effectively start the remaining oil in the middle after flowing through the water flooding range. This leads to the fact that if the water flooding range is included in the calculation of the overall flooding effect, the lifting effect of all three stage chemical slugs is not obvious.

**Figure 6:**
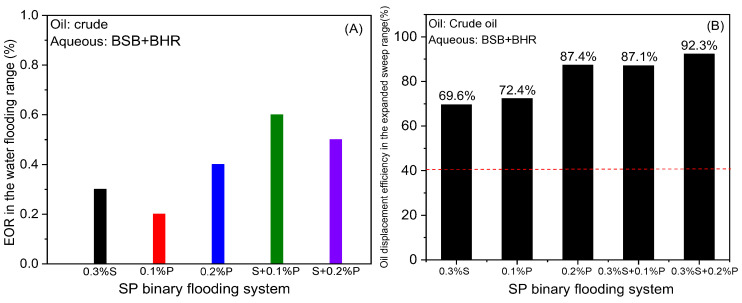
(**A**) Enhanced oil displacement efficiency in the range of water flooding. (**B**) Oil displacement efficiency in the added sweep range after S/P/SP system flooding. The red dotted line in (**B**) represents the average oil displacement efficiency of water flooding.

To clearly reflect the lifting effect of the three stage chemical slugs, we carried out a detailed analysis of the oil displacement effect according to different regions. The improvement effect is quantified from two perspectives: the water flooding range and the expanded area outside the water flooding range. Figure 6A shows the change in oil displacement efficiency in the range of water flooding after S/P/SP flooding. It can be obviously found that the five groups of displacement fluids cannot effectively mobilize the water flooding residual oil when flowing through the water flooding range, and the increase in oil displacement efficiency is less than 1.0%. However, in the expanded area outside the water flooding range, the surfactant solution with ultra-low IFT improves the ability to start crude oil and the oil washing efficiency is significantly improved. The high viscosity of polymer solution produces higher fluid resistance, which provides higher energy to start crude oil, and the oil washing efficiency is also markedly increased. What is more, the SP system (0.3% S + 0.2% P) not only reduces the mobility of the injected fluid but also exerts the profile control effect and starts the residual oil by low IFT [34]. On the whole, 0.3% S + 0.2% P flooding demonstrated both the sweep effect and high oil washing efficiency. The efficiency in this area can be as high as 92.3%, thus recovering more crude oil, which is 51.5% higher than that of water flooding (40.8%) in Figure 6B. In addition, it can be more obvious that the three stage chemical slugs have a better displacement effect on crude oil in the un-started area of water flooding.

### 2.4. Oil Displacement Mechanism of SP Binary System

**Figure 7:**
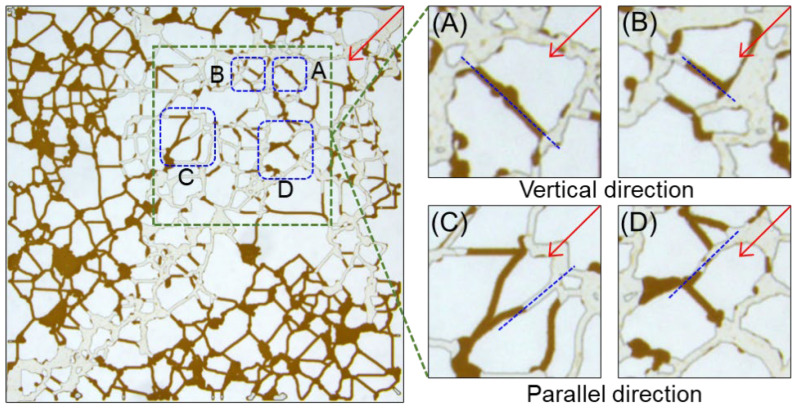
The remaining oil state after water flooding: vertical directions (**A**,**B**); parallel directions (**C**,**D**).

Figure 7 analyzes the distribution of residual oil after water flooding. The diagram of oil displacement effect obtained in the experiment was extracted from the middle of the water flooding channel into four analysis areas. They are classified as vertical (Figure 7A,B) and parallel (Figure 7C,D) to the displacement direction. For water flooding, the displacement rate of formation water along the diagonal direction is faster, which is dominated by viscous fingering and forms the mainstream area [35]. When the crude oil in the throat is perpendicular to the displacement direction, the shear force on the crude oil is weak. The formation water cannot effectively activate the crude oil at this location, leaving a high amount of columnar, porous, and clustered residual oil. In this context, the classification standard of residual oil is consistent with that defined by Wang et al. [36]. Parallel to the displacement direction, even in the mainstream dominant pathway, porous, and columnar oil also remain. To clearly present the oil recovery effect in the expanded area outside the water flooding range, we identify the residual oil in the water flooding range and the expanded area outside the water flooding range, and count the clustered, porous, and columnar oil in Table 2.

As a whole, the P/SP solutions can effectively activate large oil clusters (cluster and porous) in the area expanded beyond the water flooding range, and the number of remaining oil clusters is noticeably reduced compared with water flooding, as shown in Table 2. Then, the large oil clusters are gradually broken down into small columnar oils, resulting in an increase in the number of columnar remaining oil. For BSB flooding, ultra-low IFT can be easily achieved between BSB solution and crude oil alone. However, most of the solution only flows along the dominant channel of water flooding due to the low viscosity of the fluid, which will not cause a pressure rise. Slight emulsification with crude oil to form an O/W emulsion increased the oil washing efficiency, but the overall oil recovery efficiency was still very low (41.5%), only 0.9% higher than water flooding. Simultaneously, since it did not expand in the newly added area, no clustered and porous residual oil appeared in this area.

After the polymer solution is injected, the molecular conformation of the polymer changes under the shear action in the porous medium. The polymer chains pass through the pore throat structure by curling, stretching, and transitioning, coupled with shear thickening behavior observed [37]. It not only effectively reduces the mobility ratio and increases the sweep range but also adsorbs and retains in the pores, blocking large pores and causing the pressure to rise. These improve the mobilization effect of residual oil not swept by water flooding in low permeability layers and displace the trapped crude oil from the pores [38]. However, from the perspective of the expanded sweep range, some porous and columnar oils are left in the area, and the remaining oil state is also similar to that of water flooding.

**Figure 8:**
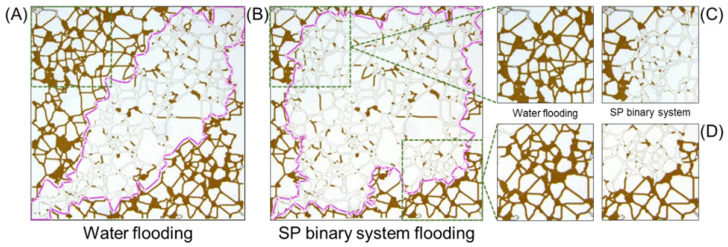
The oil displacement effect of (**A**) water flooding and (**B**) SP binary system flooding on the remaining oil; (**C**,**D**) two analysis areas were extracted within the expanded range of SP system, which were after water and SP binary system flooding.

Taking the 0.3%S + 0.2%P system as an example, the changes in the residual oil are shown in detail. The sweep range in Figure 8A,B shows that the SP system (0.3%S + 0.2%P) can significantly expand the sweep range based on water flooding. Upon closer examination, the remaining oil state within the water flooding range has not changed. In the extended sweep range, two analysis areas (corner of the model), were extracted, as shown in Figure 8C,D. It can be found that there are almost no large clusters of residual oil in this area. All of them have been activated, emulsified, peeled off, and gradually decomposed into a certain number of small discontinuous oil clusters (Table 2). This also shows that the SP solution mobilizes more residual oil and can effectively improve the oil washing efficiency in the expanded swept area, which is obviously better than that of formation water, surfactant, and polymer flooding. These oil displacement effects are supported by the parameters (IFT and bulk viscosity) obtained from the macroscopic interface property test experiments, which verify the correctness of the conclusion that SP synergy can significantly improve oil recovery and microscopic oil displacement mechanism in micro-scale experiments.

## 3. Materials and Methods

### 3.1. Materials

**Figure 9:**
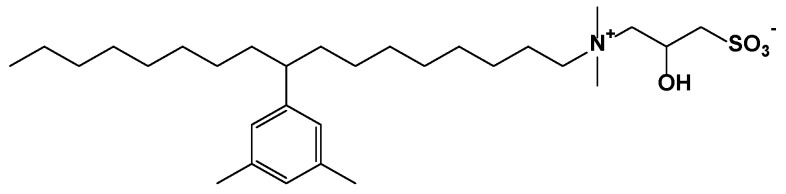
Structure and abbreviation of BSB.

**Figure 10:**
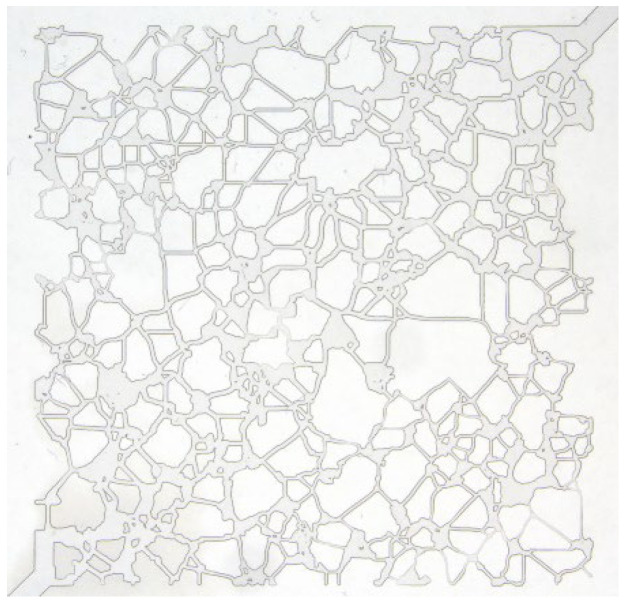
Shape and structure of microfluidic chip model.

The zwitterionic surfactant betaine (BSB) used in this study comes from the China Petroleum Exploration and Development Research Institute, with a purity of 95%, and its structure and abbreviation are shown in Figure 9. The crude oil was taken from the Qinghai Oilfield in China, with a viscosity of 28.30 cP at 25 °C and a viscosity of 4.36 cP at reservoir temperature (120 °C). The contents of saturates, aromatics, resins, and asphaltenes in crude oil are 75.97%, 11.89%, 9.56%, and 2.58%, respectively. A new type of hydrophobically modified polyacrylamide (BHR), modified by 2-acrylamide-2-methyl propyl sulfonic acid (AMPS) for temperature- and salinity-resistance, is also provided by PetroChina Research Institute of Petroleum Exploration and Development. The molecular weight is 1980 × 10^4^ and the degree of hydrolysis is 13.2%. The composition of simulated formation water is shown in Table 3, and all the displacement solutions used in this paper are prepared by simulated formation water. The chip model used in the visualization oil displacement experiments was provided by China University of Petroleum (Beijing). The model is water-wet type, and its shape is shown in Figure 10. In this case, the side length is 15.00 × 15.00 mm; the throat width is 50~80 μm; the hole diameter is 200~800 μm; the pore depth is 0.03 mm; the pore area is 52.53 mm^2^; and the pore volume is 1.58 mm^3^.

### 3.2. Apparatus and Method

#### 3.2.1. Interfacial Tension Test

A spinning drop interfacial tensiometer (TX-500C) (Beijing Shengwei Technology Co., Ltd., Beijing, China) was used to determine the dynamic interfacial tension between the oil displacement solutions and crude oil. The rotation speed was 5000 r/min and the temperature was 25 °C. The inner phase was crude oil, and the outer phase was the oil displacement agent solution. The measurement error of the IFT value was lower than ± 5%.

#### 3.2.2. Bulk Viscosity Test

Bulk viscosity tests of surfactant, polymer, and SP binary flooding solutions were performed using a rotary rheometer HAAKE MARS II (Thermo Fisher, Karlsruhe, Germany), at 25 °C. The rotor type used was C60/1^◦^Ti, and the speed was fixed at 7.34 1/s [23]. The bulk viscosity value is was the average of three repeated measurements, and the experimental error was ±0.1 cP.

#### 3.2.3. Microscopic Visualization Oil Displacement Experiment

The oil displacement experiments were completed in the chip model of the microscopic visual oil displacement system (VMF100, Eastern-Dataphy Instruments, Beijing, China) at 25 °C [10]. First, the crude oil is completely saturated into the model. Then, a microfluidic injection pump (FLOW-EZ S/N: 12490) was used to inject the displacement fluid from the upper right inlet at a constant injection rate of 0.1 μL/min, and the displaced fluid was collected from the lower left outlet. Among them, water flooding was injected to 1 pore volume (PV), followed by injection of the oil displacement agent solution for 2 PV. The displacement process of the whole binary compound flooding system was recorded using a microscope. Results from a representative experiment are shown.

## 4. Conclusions

In this paper, the synergistic effect of the oil displacement mechanism between betaine BSB and hydrophobically modified polyacrylamide BHR was explored. The main experimental results are summarized as follows:

(1) For water flooding, due to the high water/oil mobility ratio, the formation water breaks through the crude oil along the diagonal direction, dominated by the viscous fingering phenomenon, and forms the mainstream area. Only a small amount of crude oil is displaced, resulting in a low average sweep efficiency (48.4%) and oil displacement efficiency (about 40%).
(2) The S/P/SP solution has a weak ability to mobilize the remaining oil in the water flooding range, but it can play an excellent displacement effect in the expanded area outside the water flooding range. Therefore, for this type of reservoir, we recommend the use of chemical flooding in the initial stage rather than after water flooding.
(3) In the newly affected area outside the water flooding range, the surfactant solution with ultra-low IFT improves the ability to mobilize crude oil, and the oil washing efficiency is obviously improved. The high viscosity of the polymer solution produces higher fluid resistance, improving the energy required to mobilize crude oil, and the oil washing efficiency is also significantly increased. The SP system (0.3% S + 0.2% P) not only reduces the mobility of the injected fluid, but also exerts the profile control effect, and mobilizes the residual oil at low tension.
(4) The microscopic mechanism of SP binary flooding includes two aspects. The polymer increases viscosity under the shear stress of porous media, which reduces the fluid migration rate, increases pressure, and effectively expands the sweep range. In addition, the solution relies on low IFT to emulsify and mobilize the residual oil after water flooding, which can effectively decompose the large remaining oil in the swept range into discontinuous small oil clusters and increase the oil washing efficiency. The synergy of the two can greatly improve the oil displacement efficiency.

## Figures and Tables

**Table 1:** Oil displacement efficiency data of surfactant/polymer/SP binary flooding solutions.

Oil Displacement System	IFT /mN·m^−1^	Bulk Viscosity /cP	Water Flooding /%	S/P/SP Flooding /%	Oil Displacement Efficiency /%
0.3% S	1.2 × 10^−3^	1.1	40.6	41.5	0.9
0.1% P	/	7.4	40.8	46.1	5.3
0.2% P	/	22.1	41.9	53.8	11.9
0.3% S + 0.1% P	1.5 × 10^−3^	6.0	40.9	54.2	13.3
0.3% S + 0.2% P	4.3 × 10^−4^	18.6	39.6	70.9	31.3

**Table 2:** Classification and statistics of remaining oil types.

System	Water Flooding			New Affected Area		
	Cluster	Porous	Columnar	Cluster	Porous	Columnar
0.3% S	4	6	35	0	0	2
0.1% P	3	7	40	0	2	12
0.2% P	4	7	37	0	1	23
0.3% S + 0.1% P	4	3	37	0	1	12
0.3% S + 0.2% P	4	2	36	0	1	30

**Table 3:** Formulation and composition of simulated formation water.

TDS	Na^+^	Ca^2+^	Mg^2+^	Cl^−^	SO_4_^2−^	HCO_3_^−^
(mg/L)	(mg/L)	(mg/L)	(mg/L)	(mg/L)	(mg/L)	(mg/L)
145,265.75	53,574.06	1766.17	919.12	87,071.70	1444.00	490.70

## Data Availability

No new data were created or analyzed in this study. Data sharing is not applicable to this article.

## References

[1] Omari A., Cao R., Zhu Z., Xu X. (2021). A comprehensive review of recent advances on surfactant architectures and their applications for unconventional reservoirs. J. Pet. Sci. Eng..

[2] Liu T., Jin X., Wang M.R. (2018). Critical resolution and sample size of digital rock analysis for unconventional reservoirs. Energies.

[3] Wang X.Z., Peng X.L., Zhang S.J., Du Z.W., Zeng F.H. (2018). Characteristics of oil distributions in forced and spontaneous imbibition of tight oil reservoir. Fuel.

[4] Zulkifli N.N., Mahmood S.M., Akbari S., Manap A.A.A., Kechut N.I., Elrais K.A. (2020). Evaluation of new surfactants for enhanced oil recovery applications in high-temperature reservoirs. J. Pet. Explor. Prod. Technol..

[5] Liu R., Du D.J., Pu W.F., Zhang J., Fan X.B. (2018). Enhanced oil recovery potential of alkyl alcohol polyoxyethylene ether sulfonate surfactants in high-temperature and high-salinity reservoirs. Energy Fuels.

[6] Lu J., Goudarzi A., Chen P., Kim D.H., Delshad M., Mohanty K.K., Sepehrnoori K., Weerasooriya U.P., Pope G.A. (2014). Enhanced oil recovery from high-temperature, high-salinity naturally fractured carbonate reservoirs by surfactant flood. J. Pet. Sci. Eng..

[7] Mandal A. (2015). Chemical flood enhanced oil recovery: A review. Int. J. Oil Gas Coal Technol..

[8] Pu W.F., Yuan C.D., Hu W.G., Tan T., Hui J., Zhao S., Wang S., Tang Y.L. (2016). Effects of interfacial tension and emulsification on displacement efficiency in dilute surfactant flooding. RSC Adv..

[9] Al-Azani K., Abu-Khamsin S., Al-Abdrabalnabi R., Kamal M.S., Patil S., Zhou X., Hussain S.M.S., Al Shalabi E. (2022). Oil recovery performance by surfactant flooding: A perspective on multiscale evaluation methods. Energy Fuels.

[10] Yu F.W., Jiang H.Q., Fan Z., Xu F., Su H., Cheng B.Y., Liu R.J., Li J.J. (2019). Features and imbibition mechanisms of Winsor I type surfactant solution in oil-wet porous media. Petrol. Explor. Dev..

[11] Su H., Zhou F.J., Liu Y., Gao Y.J., Cheng B.Y., Dong R.C., Liang T.B., Li J.J. (2021). Pore-scale investigation on occurrence characteristics and conformance control mechanisms of emulsion in porous media. Petrol. Explor. Dev..

[12] Li X.X., Yue X.A., Zou J.R., Yan R.J. (2022). Effect of in-situ emulsification of surfactant on the enhanced oil recovery in low-permeability reservoirs. Colloids Surf. A.

[13] Sun Z., Wu X.C., Kang X.D., Lu X.G., Li Q., Jiang W.D., Zhang J. (2019). Comparison of oil displacement mechanisms and performances between continuous and dispersed phase flooding agents. Petrol. Explor. Dev..

[14] Amoyav B., Benny O. (2019). Microfluidic based fabrication and characterization of highly porous polymeric microspheres. Polymers.

[15] Wever D.A.Z., Picchioni F., Broekhuis A.A. (2011). Polymers for enhanced oil recovery: A paradigm for structure–property relationship in aqueous solution. Prog. Polym. Sci..

[16] Khorsandi S., Qiao C., Johns R.T. (2017). Displacement efficiency for low salinity polymer flooding including wettability alteration. SPE J..

[17] Cao H., Li Y.Q., Gao W.B., Cao J.X., Sun B.Y., Zhang J. (2023). Experimental investigation on the effect of interfacial properties of chemical flooding for enhanced heavy oil recovery. Colloids Surf. A.

[18] Babu K., Pal N., Bera A., Saxena V.K., Mandal A. (2015). Studies on interfacial tension and contact angle of synthesized surfactant and polymeric from castor oil for enhanced oil recovery. Appl. Surf. Sci..

[19] Lacey M., Hollis C., Oostrom M., Shokri N. (2017). Effects of pore and grain size on water and polymer flooding in micromodels. Energy Fuels.

[20] Zhang Z.Y., Wang Y.F., Ding M.C., Mao D.H., Chen M.F., Han Y.G., Liu Y.G., Xue X.F. (2023). Effects of viscosification; ultra-low interfacial tension, and emulsification on heavy oil recovery by combination flooding. J. Mol. Liq..

[21] Panthi K., Weerasooriya U., Mohanty K.K. (2020). Enhanced recovery of a viscous oil with a novel surfactant. Fuel.

[22] Lv W.F., Zhou Z.H., Zhang Q., Zhang X.J., Zhang L. (2023). Study on the mechanism of surfactant flooding: Effect of betaine structure. Adv. Geo-Energy Res..

[23] Sun Q., Hu F.T., Han L., Zhu X.Y., Zhang F., Ma G.Y., Zhang L., Zhou Z.H., Zhang L. (2023). The synergistic effects between sulfobetaine and hydrophobically modified polyacrylamide on properties related to enhanced oil recovery. Molecules.

[24] Zhang Q., Zhan S.Y., Zhou Z.H., Li Z.X., Lu W.H., Zhang L., Zhu Y., Zhang L. (2017). Effect of crude oil fractions on the interfacial tensions of alkali-betaine mixed solutions. J. Pet. Sci. Eng..

[25] Cao J.H., Zhou Z.H., Xu Z.C., Zhang Q., Li S.H., Cui H.B., Zhang L., Zhang L. (2016). Synergism/antagonism between crude oil fractions and novel betaine solutions in reducing interfacial tension. Energy Fuels.

[26] Ma B.D., Gao B.Y., Zhang L., Gong Q.T., Jin Z.Q., Zhang L., Zhao S. (2014). Influence of polymer on dynamic interfacial tensions of EOR surfactant solutions. J. Appl. Polym. Sci..

[27] Xin X., Xu G.Y., Gong H.J., Bai Y., Tan Y.B. (2008). Interaction between sodium oleate and partially hydrolyzed polyacrylamide: A rheological study. Colloids Surf. A.

[28] Zhang L.J., Yue X.A. (2008). Displacement of polymer solution on residual oil trapped in dead ends. J. Cent. South Univ. Technol..

[29] Algharaib M., Alajmi A., Gharbi R. (2014). Improving polymer flood performance in high salinity reservoirs. J. Pet. Sci. Eng..

[30] Zhang L.J., Yue X.A., Guo F.Q. (2008). Micro-mechanisms of residual oil mobilization by viscoelastic fluids. Pet. Sci..

[31] Taylor K.C., Nasr-El-Din H.A. (1998). Water-soluble hydrophobically associating polymers for improved oil recovery: A literature review. J. Pet. Sci. Eng..

[32] Lu C.H., Jiang H.Q., You C.C., Wang Y., Ma K., Li J.J. (2021). A novel method to determine the thief zones in heavy oil reservoirs based on convolutional neural network. J. Pet. Sci. Eng..

[33] Sun Q., Zhou Z.H., Han L., Zou X.Y., Qiao L.G., Zhang Q., Zhang F., Zhang L., Zhang L. (2023). How to regulate the migration ability of emulsions in micro-scale pores: Droplet size or membrane strength?. Molecules.

[34] Tan F.Q., Ma C.M., Qin J.H., Li X.K., Liu W.T. (2022). Factors influencing oil recovery by surfactant–polymer flooding in conglomerate reservoirs and its quantitative calculation method. Pet. Sci..

[35] Mohammadzadeh O., Sedaghat M.H., Kord S., Zendehboudi S., Giesy J.P. (2019). Pore-level visual analysis of heavy oil recovery using chemical-assisted waterflooding process—Use of a new chemical agent. Fuel.

[36] Wang C., Jiang H.Q., Liu J.W., Mi L.D., Li J.J. An advanced approach to study the seepage characteristics of dynamic remaining oil in porous media at pore scale. Proceedings of the SPE Europec Featured at 79th EAGE Conference and Exhibition.

[37] Lewandowska K. (2007). Comparative studies of rheological properties of polyacrylamide and partially hydrolyzed polyacrylamide solutions. J. Appl. Polym. Sci..

[38] Abdelgawad K.Z. (2022). Polymer induced permeability reduction: The influence of polymer retention and porous medium properties. J. Pet. Sci. Eng..

